# The Immunomodulatory Effect of Silver Nanoparticles in a Retinal Inflammatory Environment

**DOI:** 10.1007/s10753-024-02128-w

**Published:** 2024-08-27

**Authors:** Katerina Palacka, Barbora Hermankova, Tereza Cervena, Pavel Rossner, Alena Zajicova, Eva Uherkova, Vladimir Holan, Eliska Javorkova

**Affiliations:** 1 Department of Toxicology and Molecular Epidemiology, Institute of Experimental Medicine of the Czech Academy of Sciences, 142 20 Prague 4, Czech Republic; 2 Department of Cell Biology, Faculty of Science, Charles University, 128 43 Prague 2, Czech Republic; 3 Department of Ophthalmology, First Faculty of Medicine, Charles University and General University Hospital, 121 08 Prague 2, Czech Republic

**Keywords:** Retinal diseases, Microglia, Neuroinflammation, Neovascularization, Silver nanoparticles

## Abstract

Activation of immune response plays an important role in the development of retinal diseases. One of the main populations of immune cells contributing to the retinal homeostasis are microglia, which represent a population of residential macrophages. However, under pathological conditions, microglia become activated and rather support a harmful inflammatory reaction and retinal angiogenesis. Therefore, targeting these cells could provide protection against retinal neuroinflammation and neovascularization. In the recent study, we analyzed effects of silver nanoparticles (AgNPs) on microglia in vitro and in vivo. We showed that the AgNPs interact in vitro with stimulated mouse CD45/CD11b positive cells (microglia/macrophages), decrease their secretion of nitric oxide and vascular endothelial growth factor, and regulate the expression of genes for Iba-1 and interleukin-1β (IL-1β). In our in vivo experimental mouse model, the intravitreal application of a mixture of proinflammatory cytokines tumor necrosis factor-α, IL-1β and interferon-γ induced local inflammation and increased local expression of genes for inducible nitric oxide synthase, IL-α, IL-1β and galectin-3 in the retina. This stimulation of local inflammatory reaction was significantly inhibited by intravitreal administration of AgNPs. The application of AgNPs also decreased the presence of CD11b/Galectin-3 positive cells in neuroinflammatory retina, but did not influence viability of cells and expression of gene for rhodopsin in the retinal tissue. These data indicate that AgNPs regulate reactivity of activated microglia in the diseased retina and thus could provide a beneficial effect for the treatment of several retinal diseases.

## Background

Retinal microglia play an important role in maintaining tissue homeostasis and in the production of several factors, such as nerve growth factor (NGF), glial cell derived neurotrophic factor (GDNF) and basic fibroblast growth factor (bFGF), which are important for retinal cell survival [1, 2]. However, under chronic pathological conditions, including diabetic retinopathy (DR), age-related macular degeneration (AMD) and glaucoma, the activation of microglia contributes to the progression of these diseases. Microglia can be divided according to their phenotype into anti-inflammatory and proinflammatory types [3]. In the degenerated retina, activated microglia accumulate in the tissue and contribute to the apoptosis of retinal cells. In the proinflammatory state, microglia enhance the expression of CD45 and Iba-1 markers and increase the production of several factors such as interleukin-1β (IL-1β), tumor necrosis factor- α (TNF-α) and nitric oxide (NO). Activated microglia contribute to the breaking of the blood-retinal barrier and to the development of retinal pathology [3–5]. In addition, it has been shown that an increased number of microglia persist in the degenerated retina, and the local chronic inflammation state is associated with increased production of TNF-α, IL-1β and interferon-γ (IFN-γ) [6]. It was also shown that cytokines of IL-1 family play an important role in retinal degenerative diseases. These cytokines are produced by several retinal cell types, including microglia, and play a role in macrophage accumulation, secretion of other proinflammatory cytokines, amplification of the inflammatory response and the induction of cell death. Thus, the regulation of IL-1β secretion has been suggested as an effective tool for amelioration of retinal pathology [7]. Retinal inflammation is also associated with increased expression of galectin-3 [8, 9]. Galectin-3 is a lectin that has several functions including regulation of an inflammatory response and it was demonstrated that galectin-3 is also produced by activated retinal microglia [10]. Moreover, it was suggested that galectin-3 is responsible for microglia activation and for the induction of neurotoxicity. Therefore, therapy focusing on this lectin could be a promising option for the treatment of several retinal pathologies [11, 12].

Currently, treatment options for retinal degenerative diseases are very limited and are associated with several complications. Procedures, such as laser therapy or intravitreal applications of drugs only slow down the progression of diseases and are not suitable for all patients. Thus, novel therapeutic approaches remain to be the subject of investigation.

Nanoparticles (NPs, particles with a size of 100 nm or less) are widely used in medicine as drug delivery vehicles, antibacterial components and diagnostic tools [13]. NPs can be derived from various natural or synthetic sources. For treatment of ocular diseases, NPs have been considered for several reasons. It has been shown that NPs exert antioxidative, anti-angiogenic and anti-inflammatory properties, depending on their source, size and doses [14]. For example, an anti-angiogenic effect of titanium dioxide nanoparticles (TiO_2_NPs) and silicate nanoparticles (SiNPs) was observed in oxygen-induced retinopathy [15, 16]. In addition, it was shown that intravitreal administration of both types of NPs did not cause cytotoxicity. Similar results were also obtained with the application of gold nanoparticles (AuNPs) [17]. In another study, cerium oxide nanoparticles (CeO_2_NPs), which are known for their antioxidative properties, reduced the activation of microglia in the retina following light-induced damage [18]. Silver nanoparticles (AgNPs) are widely used in various studies for their antibacterial properties [13]. It has also been shown, that AgNPs inhibit the production of VEGF and IL-1β in retinal endothelial cells [19] and thus could be used in anti-inflammatory and anti-angiogenic treatment [20].

In our study, we investigated the possible inhibitory effect of AgNPs on immune response in the retina and on the regulation of microglia activity. The results showed that a local application of AgNPs reduces the inflammatory reaction in the retina, and thus AgNPs could be considered as a promising tool for the treatment of retinal diseases.

## Methods

### Preparation of AgNPs

AgNPs were purchased from Sigma-Aldrich (St. Louis, MO, USA) as an uncoated silver nanopowder. The preparation of AgNP stock dispersions was described in detail elsewhere [21]. AgNPs were sonicated, dispersed to a final concentration of 2.56 mg/ml and diluted in culture medium for the required concentrations used in the experiments.

### Animals

Female mice of inbred strain BALB/c (at the age of 8–15 weeks) obtained from the Institute of Molecular Genetics of the Czech Academy of Sciences, Prague or purchased from Envigo company (Indianapolis, IN, USA), were used in the experiments. The use of animals was approved by the Local Ethical Committee of the Institute of Experimental Medicine of the Czech Academy of Sciences, Prague (approval code 6130/2022).

### The Cultivation of Retinal Explants in the Presence of AgNPs and Proinflammatory Cytokines

For in vitro experiments, retinas were isolated from untreated mice. The eyeball was enucleated from euthanized mice, and extraocular muscle, connective tissue, cornea, lens and vitreous were removed. The retina was gently discharged from the remaining posterior eyeball and placed into a 48-well tissue culture plate (Techno Plastic Products, TPP, Trasadingen, Switzerland) with 1 ml of RPMI 1640 medium (Sigma-Aldrich), containing 10% of fetal bovine serum (FBS, Gibco BRL, Grand Island, NY, USA), 100 U/ml of penicillin, 100 µg/ml of streptomycin and 10 mM HEPES buffer (referred to as complete RPMI 1640 medium).

Retinal explants were cultivated alone or in the presence of 25 μg/ml or 50 μg/ml AgNPs. After 48 h, the metabolic activity was analyzed by WST-1 test (according to manufacturer’s instruction). Retinal explants were cultivated either unstimulated, in the presence of AgNPs (the final concentration of 25 μg/ml was selected based on our preliminary metabolic activity WST-1 test), in the presence of proinflammatory cytokines TNF-α, IL-1β and IFN-γ (PeproTech, Rocky Hill, NJ, USA) each in a final concentration of 10 ng/ml, or in the presence of cytokines and AgNPs.

After a 48-h cultivation, retinas were transferred into 500 µl of TRI reagent (Molecular Research Centre, Cincinnati, OH, USA) and stored at -80 °C.

### Isolation of Bone Marrow Cells and Preparation of the CD45/CD11b Population

Femurs and tibias were isolated from BALB/c mice, and bone marrow was flushed out and homogenized using a tissue homogenizer. Single cell suspensions were seeded in a 75-cm^2^ tissue culture flask (TPP) in Dulbecco’s modified Eagle medium (DMEM, Sigma-Aldrich) containing 10% FBS, antibiotics (100 U/ml of penicillin, 100 µg/ml of streptomycin) and 10 mM HEPES buffer (referred to as complete DMEM). After a 48-h incubation, non-adherent cells were washed out and the remaining adherent cells were cultivated at 37 °C in an atmosphere of 5% CO_2_ for an additional 2 weeks. The cells were harvested after the 3rd passage using 1 ml of 0.5% trypsin solution (Sigma-Aldrich), then gently scraped and incubated with CD11b and CD45 MicroBeads (Miltenyi Biotec, Bergisch Gladbach, Germany) for 15 min according to the manufacturer’s instructions. Cells were isolated using a magnetic activated cell sorter (Auto-MACS, Miltenyi Biotec) and a positive population (CD45/CD11b positive cells) was used in further experiments.

For phenotypic characterization, the CD45/CD11b population was seeded (1 × 10^6^ cells) in a 25 cm^2^ cultivation flask (TPP) in 5 ml of complete RPMI 1640 medium or in 5 ml of complete RPMI 1640 medium with proinflammatory cytokines TNF-α, IL-1β and IFN-γ (final concentration 10 ng/ml). After a 48-h incubation, cells were harvested and characterized by flow cytometry.

### Determination of the Effect of AgNPs on the Metabolic Activity of the CD45/CD11b Population

A test of metabolic activity was performed to determinate suitable concentrations of AgNPs used in experiments with the CD45/CD11b population. The cells were seeded in a 96-well plate (30 000 cells/well) in 200 μl of complete RPMI 1640 medium and cultured alone or in the presence of different concentrations of AgNPs (0—50 μg/ml) or cytokines TNF-α, IL-1β and IFN-γ (10 μg/ml) and AgNPs (0—50 μg/ml). After a 48-h incubation, 20 µl of water-soluble tetrazolium-1 (WST-1, Roche, Penzberg, Germany) was added into each well and incubated for an additional 2 h. The optical density was measured using Sunrise Remote ELISA reader (Gröding, Austria) at absorbance of wavelength 450 nm. Concentrations of AgNPs 3.125, 6.25 and 12.5 μg/ml were selected for additional experiments.

### Cultivation of the CD45/CD11b Population in the Presence of Proinflammatory Cytokines and AgNPs

CD45/CD11b-positive cells were seeded into a 48-well plate (200 000 cells/well) in 1 ml of complete RPMI 1640 medium with TNF-α, IL-1β and IFN-γ (10 ng/ml) and AgNPs in concentrations of 3.125, 6.25 and 12.5 μg/ml. After a 48-h cultivation, the supernatants were harvested and used for analysis of the production of NO and VEGF. Cells were transferred into 500 µl of TRI reagent and stored at -80 °C. In the 2nd set of experiments, CD45/CD11b positive cells were prestimulated with TNF-α, IL-1β and IFN-γ (10 ng/ml), and after a 48-h cultivation the cells were washed, medium was exchanged, and the cells were cultivated for an additional 48 h with AgNPs (3.125, 6.25 and 12.5 μg/ml). In both experiments, unstimulated cells were used as a control.

### Intravitreal Application of Proinflammatory Cytokines and AgNPs

Mice were anesthetized using an intraperitoneal injection of xylazine (Bioveta, Ivanovice, Czech Republic) and ketamine (Bioveta) in the ratio 1:1. The intravitreal application was performed using a Hamilton syringe (5 μl of volume, Hamilton, Reno, NV, USA) with a 33G needle (Hamilton). One group of mice received 1 μl of mixture of TNF-α, IL-1β and IFN-γ.

(each cytokine in a final concentration 10 ng/ml) injected intravitreally into the right eye. The second group received 1 μl of a mixture of TNF-α, IL-1β and IFN-γ together with 0.025 μg of AgNPs. The dose was selected based on our preliminary results. The selected dose of AgNPs did not change the viability of the retinal cells and did not change the expression of rhodopsin in the retina. Untreated mice were used as a control group. During the experiments, we did not observe any death or changes in behavior of our experimental mice.

### Enzyme-Linked Immunosorbent Assay (ELISA) and NO Detection

Concentrations of VEGF in supernatants harvested from cultures of the CD45/CD11b population were quantified using ELISA kits purchased from R&D Systems (Minneapolis, MN, USA) according to the manufacturer’s instructions. The concentration of NO was determined by Griess reaction in 100 μl of tested supernatant by adding 100 μl of 1% sulphanilamide and 0.3% N-1-naphthylenthylene diamine dihydrochloride (both in 3% H_3_PO_4_). The reaction was quantified using Sunrise Remote ELISA Reader.

### Detection of Gene Expression by RT-PCR

The total RNA was isolated from the CD45/CD11b populations or retinas by TRI reagent according to the manufacturer’s instructions. Reverse transcription was performed with deoxyribonuclease I (Promega, Madison, WI, USA) in DNase I buffer (Promega) and the first cDNA strand was synthetized with random primers (Promega) using M-MLV reverse transcriptase (Promega). The total volume of the reaction mixture was 25 μl. Quantitative RT-PCR was performed using SYBR green System (Applied Biosystems, Foster City, CA, USA) by StepOne-Plus Real-Time PCR (Applied Biosystems) with the following parameters: denaturation at 95 °C (3 min) followed by 40 cycles at 95 °C (20 s), annealing at 60 °C (30 s), elongation at 72 °C (30 s).

Fluorescence data were collected at each cycle after the elongation at 80 °C for 5 s and the collected data was analyzed by StepOne Software version 2.3 (Applied Biosystems). A comparison with the expression of the gene for glyceraldehyde 3-phosphate dehydrogenase (GAPDH) was used for the calculation of the relative expression of the analyzed gene. The primers used for amplification are shown in Table 1.

**Table 1:** Sequences of Oligonucleotides Used in RT-PCR

Gene	Forward primer	Reverse primer
*Bax*	GGTCCCGAAGTAGGAGAGGA	GTGAGCGGCTGCTTGTCT
*Bcl-2*	GTGGATGACTGAGTACCT	CCAGGAGAAATCAAACAGAG
*Galectin-3*	TGCGTTGGGTTTCACTGTGCC	GGTGCCCTATGACCTGCCCT
*GAPDH*	AGAACATCATCCCTGCATCC	ACATTGGGGGTAGGAACAC
*Iba-1*	CAGCATTCGCTTCAAGGACATA	ATCAACAAGCAATTCCTCGATGA
*IL-1α*	GAGCGCTCACGAACAGTTG	TTGGTTAAATGACCTGCAACA
*IL-1β*	AGCTGGATGCTCTCATCAGG	AGTTGACGGACCCCAAAAG
*iNOS*	TCATTGTACTCTGAGGGCTGAC	CTTTGCCACGGACGAGAC
*p53*	GTATTTCACCCTCAAGATCC	TGGGCATCCTTTAACTCTA
*Rhodopsin*	TGCCCTCAGGGATGTACC	ACCTGGATCATGGCGTTG
*VEGF*	TTTCTCCGCTCTGAACAAGG	AAAAACGAAAGCGCAAGAAA

### Preparation of Single Cell Suspension from the Retina

The isolated retina was homogenized and digested at 37 °C with 1 mg/ml of collagenase I (Sigma-Aldrich) in Hank’s balanced salt solution. Following a 45-min incubation, the digestive process was stopped by adding the excess of RPMI 1640 medium, cell suspension was filtered and washed by centrifugation.

### Flow Cytometry

Single cell suspension of the retinal cells or CD45/CD11b positive cells were incubated for 30 min at 4 °C with anti-mouse monoclonal antibodies conjugated with allophycocyanin (APC), phycoerythrin (PE) or fluorescein isothiocyanate (FITC). Antibodies were purchased from BioLegend (San Diego, CA, USA). Retinal cells were labeled with anti-CD45 (FITC, clone 30-F11), anti-CD11b (APC, clone M1/70) and anti-Galectin-3 (PE, clone Gal397) antibodies. CD45/CD11b positive cells were labeled with anti-CD45 (FITC, clone 30-F11), anti-CD11b (APC, clone M1/70), anti-CD206 (FITC, clone C068C2), anti-CD54 (FITC, clone YN1/1.7.4), anti-F4/80 (APC, clone BM8), and anti-CX3CR1 (APC, clone SA011F11) antibodies. Dead cells were stained 10 min before the flow cytometry analysis by adding Hoechst 33258 fluorescent dye (Invitrogen, Carlsbad, CA, USA). Data was collected by LSRII flow cytometer (BD Biosciences, Franklin Lakes, NJ, USA) and analyzed by FlowJo 9 (Tree Star, Ashland, OR, USA).

### Evaluation of Changes in the Ocular Fundus and Morphological Changes in the Eye

The changes in the ocular fundus was evaluated on day 7 after intravitreal applications using a modified camera system with an otoscope. For the assessment of morphological changes using hematoxylin and eosin staining, eyeballs were immersed in Tissue-Tek® O.C.T. Compound™ (SakuraFinetek, Inc., Torrance, CA, USA), and immediately frozen in 2-methyl-butane (Sigma-Aldrich, St. Louis, MO) in liquid nitrogen. Samples were cut into 7 μm thick sagittal sections and stained with hematoxylin and eosin (Vector Laboratories, Newark, CA, USA) according to a standard protocol.

### Statistical Analysis

The results are expressed as the mean + SD. Comparisons between the two groups were analyzed by Student’s t-test and multiple comparisons were performed by ANOVA. A value of p < 0.05 was considered statistically significant.

## Results

### The Effect of AgNPs on the Expression of Genes in the Retinal Explants Cultivated Alone or with Proinflammatory Cytokines

**Fig. 1:**
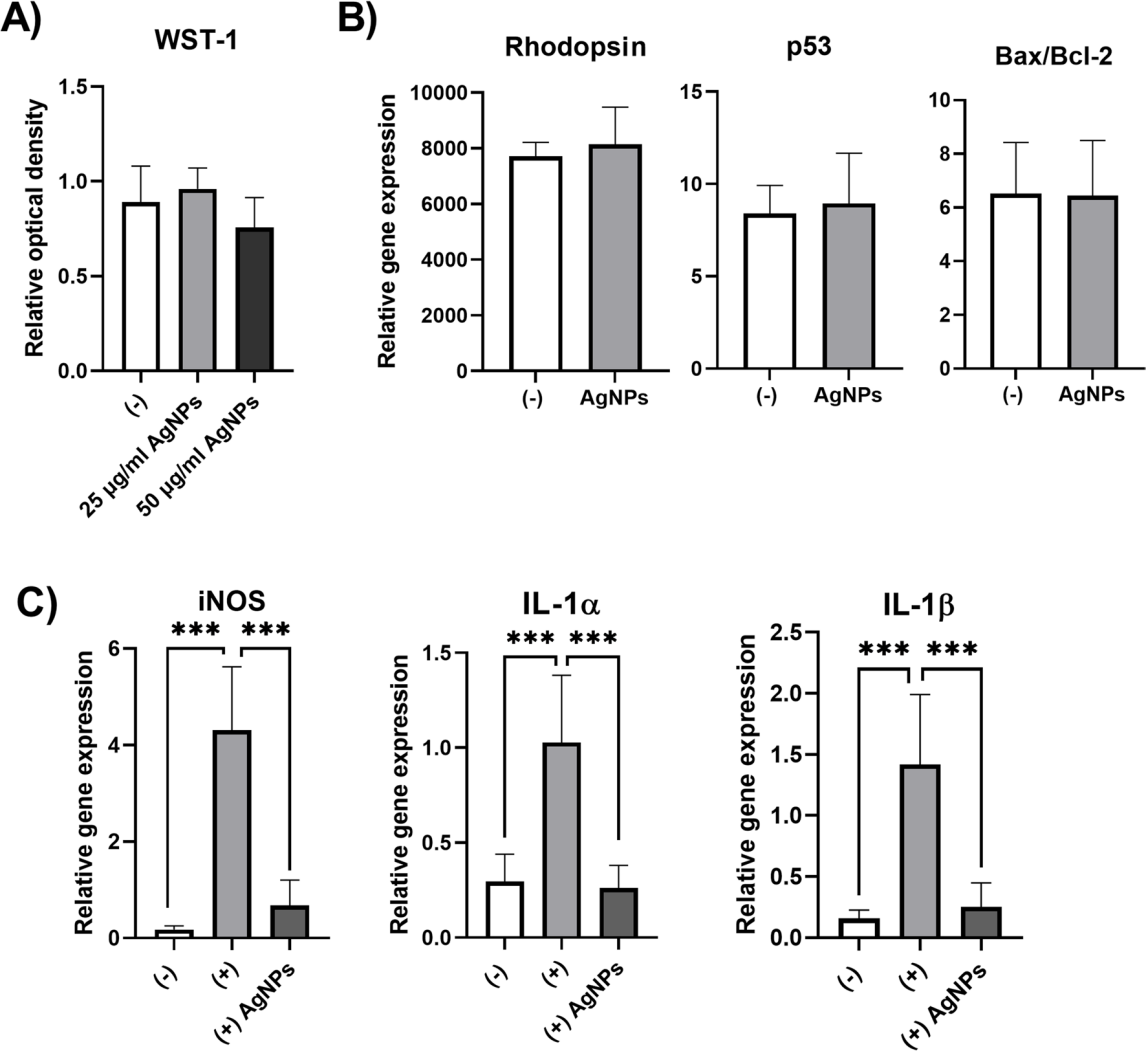
In vitro effect of AgNPs on the retinal explants cultivated alone or in the presence of proinflammatory cytokines. (**A**) The retinal explants were cultivated alone (-) or in the presence of 25 μg/ml or 50 μg/ml of AgNPs. The metabolic activity was analyzed after 48 h of cultivation. (**B**) The retinal explants were cultivated alone (-) or in the presence of 25 μg/ml of AgNPs (AgNPs). The expression of genes for rhodopsin, p53 and Bax/Bcl-2 was analyzed after a 48-h cultivation using RT-PCR. (**C**) Retinal explants were cultivated alone (-), in the presence of TNF-α, IL-1β and IFN-γ ( +) or in the presence of the cytokines and AgNPs (( +)AgNPs). The expression of genes for iNOS, IL-1α and IL-1β was analyzed after 48 h of cultivation. Each bar represents the mean + SD from three independent experiments. Asterisks represent significant difference (***p < 0.001).

The retinal explants were cultivated for 48 h in the presence of AgNPs in the concentration of 25 μg/ml or 50 μg/ml. The metabolic activity of retinal cells was measured after 48 h using WST-1 test (Fig. 1A). The selected concentration of AgNPs (25 μg/ml) did not change the expression of the gene for rhodopsin, or for genes involved in the process of apoptosis—*p53*, *Bax/Bcl-2* (Fig. 1B) but suppressed the expression of genes induced in the retina cultivated in the presence of TNF-α, IL-1β and IFN-γ (Fig. 1C). The proinflammatory cytokines increased the expression of genes for inducible nitric oxide synthase (iNOS), IL-1α and IL-1β in the retina and this increase was significantly inhibited in the presence of AgNPs.

### Phenotypic Characterization of CD45/CD11b Positive Cells Prepared from Bone Marrow

**Fig. 2:**
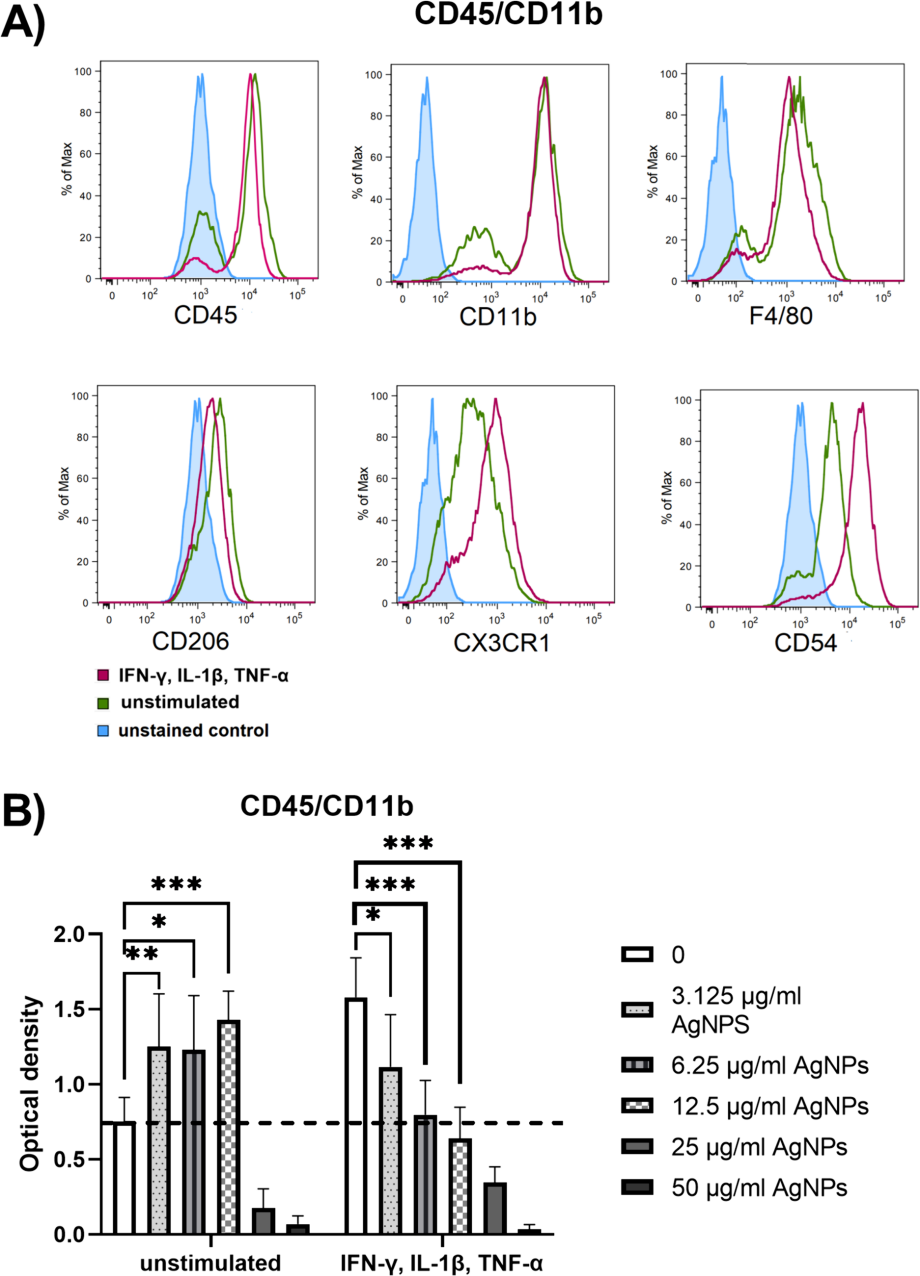
The effect of AgNPs on the viability of CD45/CD11b positive cells. (**A**) CD45/CD11b population from bone marrow isolated using magnetic activated cell sorting was cultivated unstimulated or stimulated for 48 h with proinflammatory cytokines (TNF-α, IL-1β and IFN-γ). The expression of CD45, CD11b, F4/80, CD206, CX3CR1 and CD54 markers was analyzed by flow cytometry. The histogram represents one of three similar experiments. (**B**) CD45/CD11b population was cultivated unstimulated or stimulated (TNF-α, IL-1β and IFN-γ) in the presence of various concentration of AgNPs. A test of metabolic activity was performed after 48 h of incubation by WST-1 test. Each bar represents the mean + SD from three independent experiments. Asterisks represent significant differences (*p < 0.05, **p < 0.01, ***p < 0.001).

We have described previously [22] that local administration of TNF-α, IL-1β and IFN-γ caused infiltration of immune cells (CD45^+^, CD11b^+^ and F4/80^+^) into the retina. To characterize the potential effect of AgNPs on the immune cells in the retina, a population of CD45/CD11b positive cells was isolated from bone marrow after the 3rd passage and purified by magnetic activated cell sorting. Sorted cells were cultivated for 48 h alone or in the presence of TNF-α, IL-1β and IFN-γ and characterized by flow cytometry. The cells were positive for CD45, CD11b and F4/80 indicating their microglia/macrophage phenotype. Stimulation of the cells with cytokines increased the expression of CX3CR1 and CD54 markers. A minority of cells were positive for CD206, the marker of the M2 subtype of macrophages (Fig. 2A).

### The Effect of AgNPs on the Viability and Secretory Potential of the CD45/CD11b Population

**Fig. 3:**
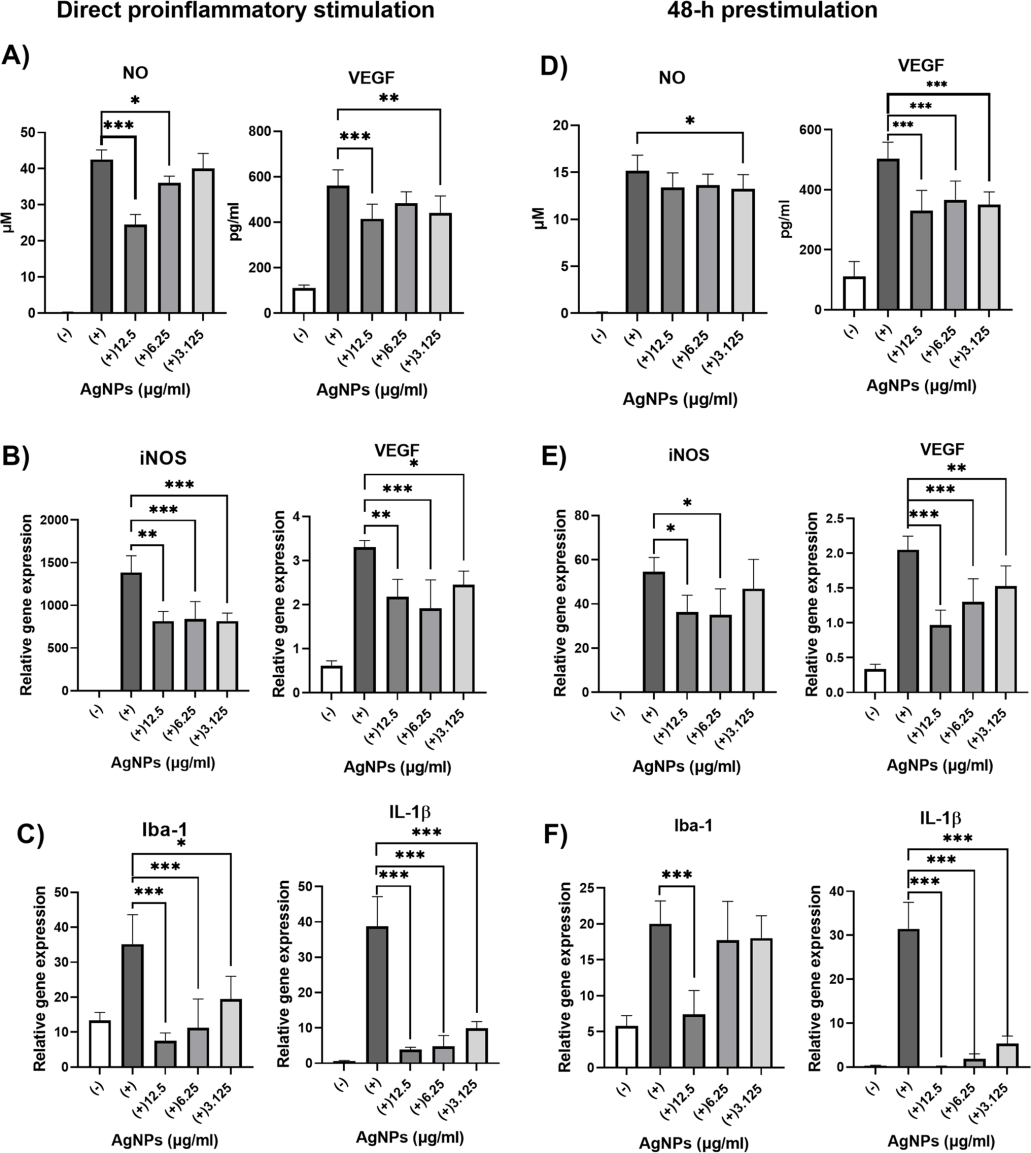
In vitro effects of AgNPs on gene expression and secretory activity of CD45/CD11b positive cells. CD45/CD11b positive cells were cultivated alone (-), stimulated with TNF-α, IL-1β and IFN-γ ( +) or cultivated with cytokines and AgNPs (12.5, 6.25, 3.125 μg/ml). (**A**) The production of NO and VEGF and (**B**) the expression of genes for iNOS and VEGF, or for (**C**) Iba-1 and IL-1β were determined after 48 h of incubation. CD45/CD11b population was prestimulated ( +) with proinflammatory cytokines. After 48 h of incubation, the cells were washed and fresh medium with AgNPs (12.5, 6.25, 3.125 μg/ml) was added for additional cultivation. (**D**) Production of NO and VEGF and (**E**) the expression of genes for iNOS, VEGF, or for (**F**) Iba-1 and IL-1β were measured after 48 h of incubation. Each bar represents the mean + SD from three independent tests. Asterisks represent significant differences (*p < 0.05, **p < 0.01, ***p < 0.001).

CD45/CD11b positive cells were cultivated with AgNPs (0—50 μg/ml) or in the presence of TNF-α, IL-1β and IFN-γ and AgNPs (0—50 μg/ml). Following a 48-h cultivation, the metabolic activity of the cells was measured by WST-1 test. Figure 2B shows that lower doses of AgNPs increased the metabolic activity of the unstimulated cells, but on the contrary decreased the metabolic activity of cells stimulated with proinflammatory cytokines. This indicates that activated CD45/CD11b positive cells are more sensitive to exposure of AgNPs than unstimulated cells, as was reported previously [23]. Concentrations of AgNPs that did not decrease cell metabolic activity in the unstimulated samples (3.125, 6.25, 12.5 μg/ml) were selected for further experiments. Stimulation of CD45/CD11b positive cells with proinflammatory cytokines increased the production of NO and VEGF, while the presence of AgNPs inhibited this effect (Fig. 3A). The result was confirmed on the gene expression level by RT-PCR (Fig. 3B). It was also shown that cells treated with AgNPs decreased the expression of the genes for Iba-1 and IL-1β (Fig. 3C). In the 2nd set of experiments, the cells were prestimulated with proinflammatory cytokines and after 48-h cultivation, exposed to AgNPs. As in the previous experiments, AgNPs inhibited the production (Fig. 3D) and the expression (Fig. 3E) of NO and VEGF and decreased the expression of the gene for IL-1β (Fig. 3F). Inhibition of the expression of *Iba-1* was observed with the highest concentration of AgNPs.

### Effects of AgNPs on CD45, CD11b and Galectin-3 Positive Cells in Retina

**Fig. 4:**
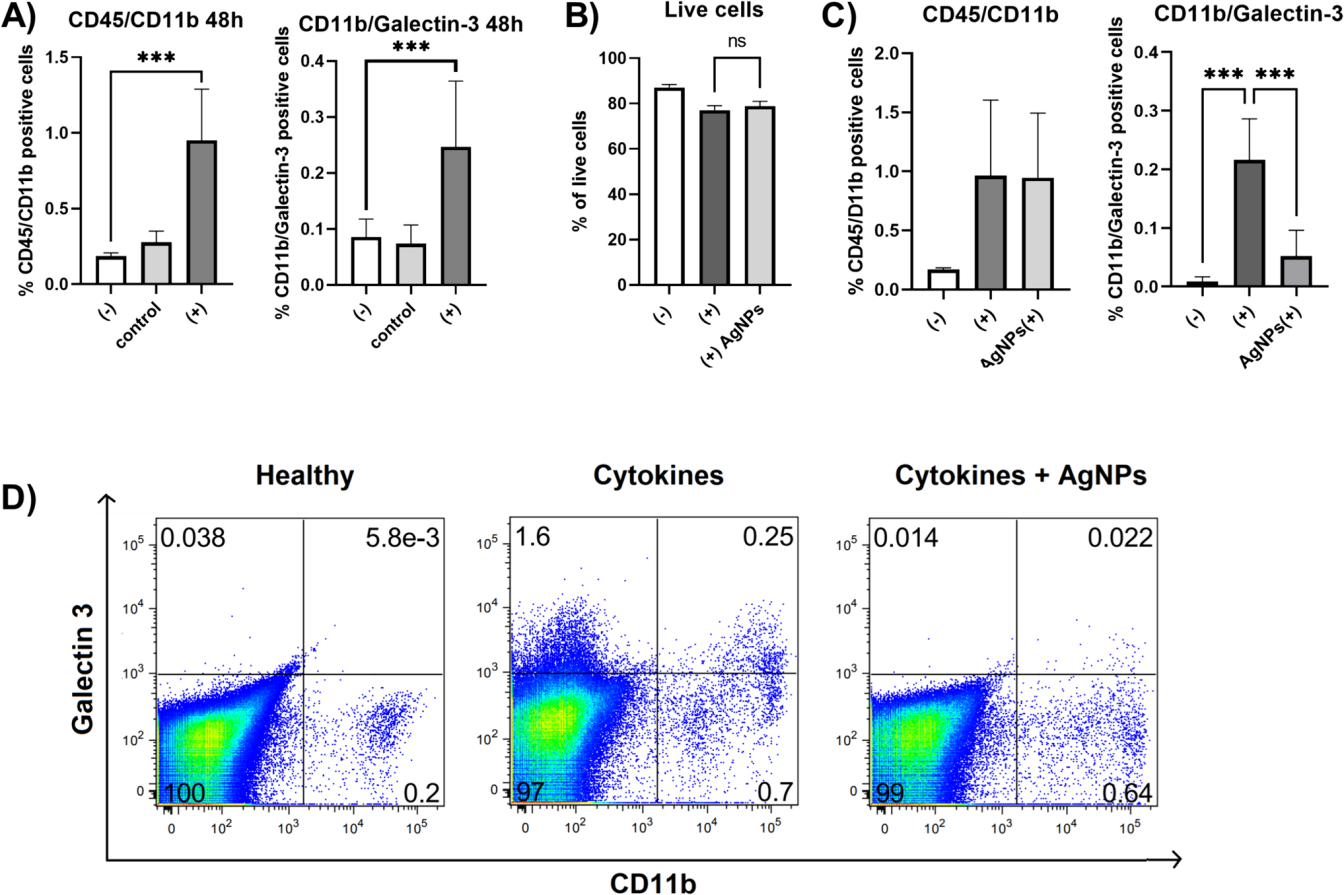
The impact of the intravitreal application of cytokines and AgNPs on the presence of CD45/CD11b and CD11b/Galectin-3 positive cells in the retina. (**A**) Mice were untreated (-), intravitreally injected with serum free medium (control) or proinflammatory cytokines TNF-α, IL-1β and IFN-γ ( +). The presence of CD45/CD11b and CD11b/Galectin-3 positive cells was analyzed 48 h after application. (**B**, **C**) Mice were untreated (-), intravitreally injected with cytokines ( +) or with cytokines and 0.025 μg AgNPs (( +)AgNPs). The percentage of live cells and of cells positive for CD45/CD11b and for CD11b/Galectin-3 were analyzed 48 h after application. (**D**) Representative dot plot of CD11b/Galectin-3 positive cells in retina. Each bar represents the mean + SD from three independent experiments. Asterisks represent significant differences (***p < 0.001).

As we have previously described [22], CD45/CD11b positive cells (microglia/macrophages) infiltrated into the retina 48 h following the intravitreal administration of TNF-α, IL-1β and IFN-γ. In addition, CD11b positive cells also increased the expression of galectin-3 (Fig. 4A). Co-application of AgNPs (0.025 μg, the dose was selected based on the preliminary results) did not influence the viability of the retinal cells (Fig. 4B) and the number of CD45/CD11b positive cells but decreased the number of CD11b/Galectin-3 positive cells (Fig. 4C,D) in comparison to the cytokine-only treated group.

### Regulation of Gene Expression in the Retina after Application of AgNPs

**Fig. 5:**
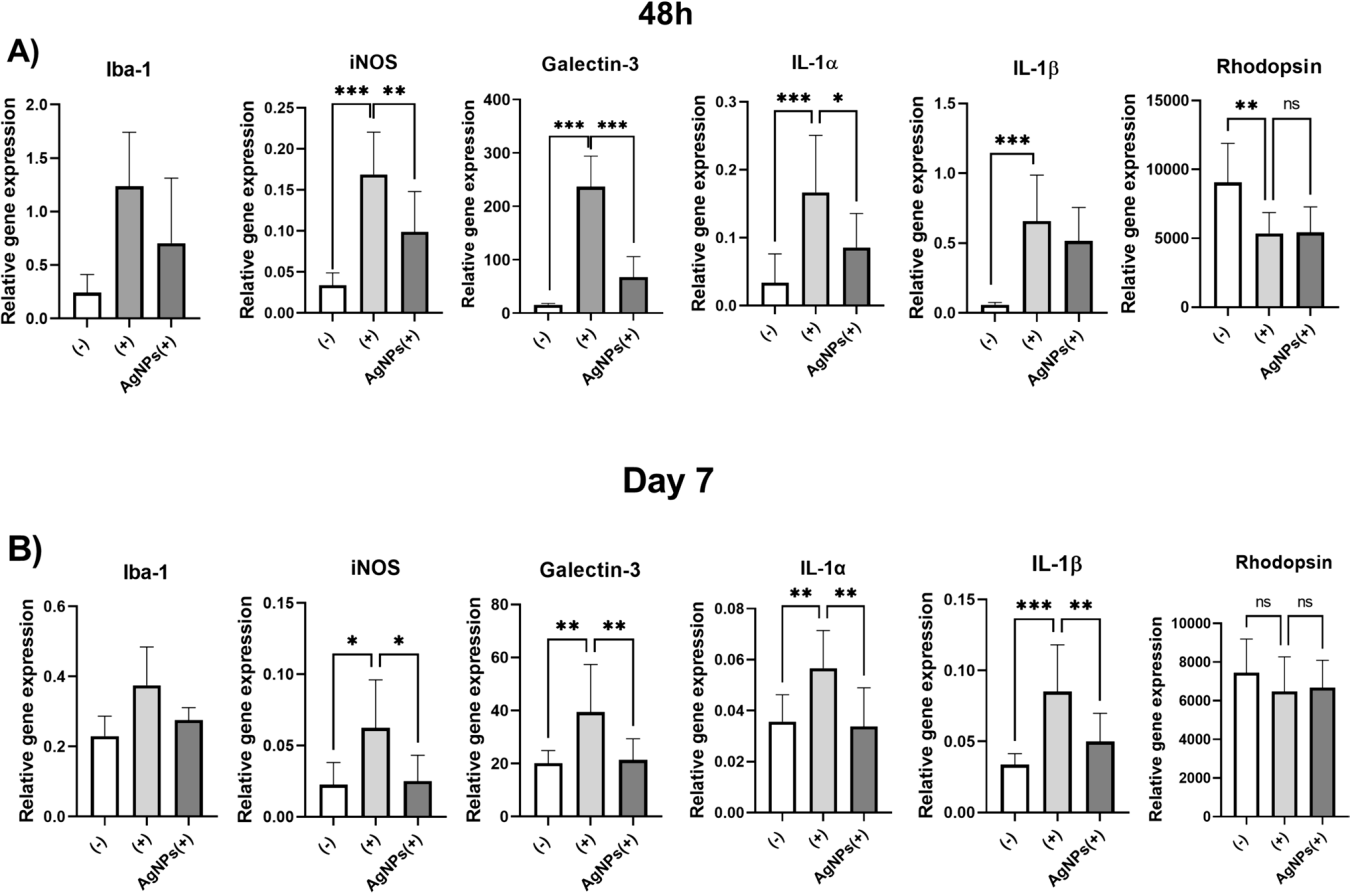
The impact of intravitreal application of AgNPs on the expression of genes in the retina. Mice were untreated (-), received intravitreal injection of proinflammatory cytokines TNF-α, IL-1β and IFN-γ ( +) or cytokines with 0.025 μg of AgNPs (( +)AgNPs). The expression of genes for Iba-1, iNOS, galectin-3, IL-1α, IL-1β and rhodopsin was analyzed (**A**) 48 h or (**B**) on day 7 after the treatment. Each bar represents the mean + SD from three independent experiments. Asterisks represent significant differences (*p < 0.05, **p < 0.01, ***p < 0.001).

The effect of AgNPs application on the gene expression in the retina was tested 48 h or 7 days after intravitreal administration. Intravitreal injection of TNF-α, IL-1β and IFN-γ increased the local expression of genes for Iba-1, iNOS, galectin-3, IL-1α and IL-1β. Simultaneous intravitreal application of proinflammatory cytokines and AgNPs significantly decreased the expression of the *iNOS*, *IL-1α* and *galectin-3* genes in both studied times (Fig. 5A, B). The reduction of the expression of the gene for IL-1β was significant on day 7 but was not significant after 48 h. The inhibition of the expression of the *Iba-1* gene was observed in both studied times. The gene expression of *rhodopsin* was decreased 48 h after intravitreal application of cytokines (Fig. 5A), but on day 7 significant differences between untreated and cytokine-treated retina were not observed (Fig. 5B). This indicates that the reduction of *rhodopsin* gene expression was a reversible reaction to the intravitreal injection. Co-application of cytokines with AgNPs did not have any effects on expression of *rhodopsin* in either studied time.

### Evaluation of Changes in the Ocular Fundus and Morphological Changes in the Eye after Intravitreal Application

**Fig. 6:**
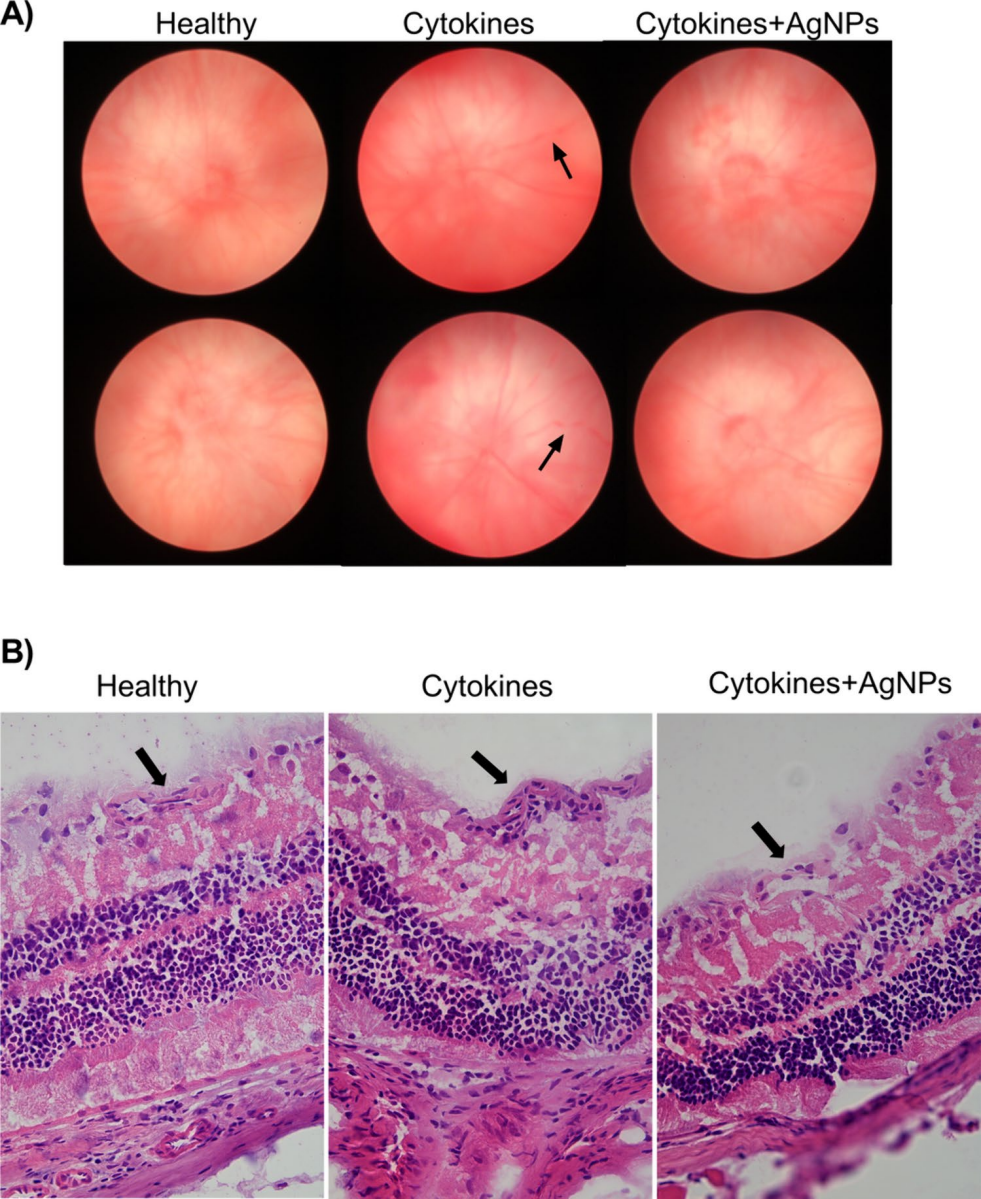
The evaluation of changes in the ocular fundus and in the morphology of the eye after intravitreal applications. (**A**) Representative images of ocular fundus. The ocular fundus was analyzed in the healthy mice, mice with applied cytokines and mice with applied cytokines and AgNPs on day 7 after local applications. Arrowheads point to disrupted blood vessels. (**B**) Representative images of the morphology of the healthy retina, retina with applied cytokines and retina with applied cytokines and AgNPs on day 7 after applications. Arrowheads point to vessels.

For the better evaluation of the therapeutic effects of AgNPs in the neuroinflammatory retina, we examined the ocular fundus of healthy mice, mice with locally applied cytokines and mice with locally applied cytokines and AgNPs. As it is shown in Fig. 6A, on day 7 after intravitreal application, proinflammatory cytokines induced dilatation and damage of blood vessels. However, the co-application of cytokines with AgNPs reduced these changes. It was also observed that the local application of cytokines is associated with the increased infiltration of blood vessel with immune cells (Fig. 6B).

## Discussion

The proinflammatory response in microglia represents one of the main pathologies among the retinal diseases. Under physiological conditions, retinal microglia remain in an anti-inflammatory state and contribute to maintaining tissue homeostasis. However, during the progression of retinal diseases, microglia produce several factors which enhance cell degeneration and decrease vision function. Thus, focusing on the regulation and attenuation of the immune response in the diseased eye could be a promising tool for ophthalmological treatment.

In recent years, NPs have been used in various fields of medicine mainly due to their unique properties. In the treatment of retinal diseases, NPs are considered for their antibacterial, anti-angiogenetic and anti-inflammatory effects [14]. Several studies using various types of NPs (SiNPs, TiO_2_NPs, AuNPs, CeO_2_NPs) showed that NPs interact with retinal microglia and attenuate angiogenesis in the inflamed retina. It has also been shown that a local application of NPs was nontoxic for retinal cells [15–18]. On the contrary, some of the studies reported that NPs displayed toxicity for retinal explants and retinal cell lines in vitro [19, 24]. These effects were dependent on the size and shapes of NPs. In our experiments, 48-h exposure of retinal explants to the selected concentration of AgNPs did not cause any significant changes in the expression of the gene for rhodopsin (marker of photoreceptors), and did not activate apoptotic genes in retinal cells. Moreover, intravitreal application of low doses (0.025 μg) of AgNPs had no significant effect on the viability of retinal cells. The observed decrease in the expression of the gene for rhodopsin was reversible and more likely associated with a reaction to intravitreal injection. We also did not observe any changes in the ocular fundus after the application of AgNPs.

In this study, we used an experimental mouse model of induction of the proinflammatory retinal environment. As we have shown previously [22], a local application of TNF-α, IL-1β and IFN-γ increased the number of immune cells in the retina and enhanced the expression of genes for proinflammatory factors. The majority of the infiltrating cells were positive for CD45 and CD11b, indicating that microglia/macrophages are the main type of immune cells playing a role in retinal inflammation. To study the effects of AgNPs on microglia/macrophages, the CD45/CD11b population was isolated from bone marrow and used in in vitro experiments. It has been previously demonstrated that cultures of bone marrow cells contain CD45/CD11b positive cells with microglia/macrophages-like characteristics [25]. In our study, the CD45/CD11b population was separated after 3 passages using magnetic activated cell sorting, and it was then characterized by flow cytometry. The cells were positive for the markers typical for microglia/macrophages which are CD45, CD11b, F4/80, CX3CR1 and CD54. A minority of cells were also positive for CD206, which is a marker of the M2 type of microglia/macrophages. The expression of CX3CR1 and CD54 was increased after stimulation with proinflammatory cytokines. Similarly, proinflammatory stimulation increased the expression of *Iba-1*, *IL-1β*, *iNOS* and *VEGF* genes and the production of NO and VEGF. This increase was also observed after medium exchange and additional cultivation, indicating that CD45/CD11b positive cells were able to remain in an activated state even after the removal of stimuli. We have reported that the presence of all used concentrations (12.5, 6.25 and 3.125 μg/ml) of AgNPs influenced the CD45/CD11b positive cells in a dose-dependent manner. For example, the expression of *Iba-1* gene (activated microglia/macrophage marker) was decreased with increasing concentrations of AgNPs, and in prestimulated cells the expression of Iba-1 was significantly decreased only by using the highest dose of NPs. On the contrary, the expression of the gene for IL-1β, a cytokine involved in several retinal diseases [7], was significantly decreased during cultivation of CD45/CD11b positive cells with all tested concentrations of AgNPs. A similar effect was observed on the gene expression and production of VEGF by CD45/CD11b positive cells. These results are in agreement with a previous study [19]. Since the regulation of VEGF production remains one of the main options in current retinal treatment [26], the application of AgNPs could provide a novel therapeutic tool for these types of pathologies.

The inhibition of *IL-1β*, *Iba-1* and *iNOS* gene expression by the application of AgNPs was confirmed in the in vivo model of the retinal proinflammatory environment. In addition, AgNPs also downregulated the expression of galectin-3 in CD11b positive cells. It was published that this lectin plays an important role in the activation of microglia and in retinal inflammation [11, 12]. Galectin-3 also enhances the pro-angiogenic activity of microglia and increases the production of VEGF [27]. Therefore, attenuation of galectin-3 could have a beneficial effect in retinal treatment, where neovascularization is one of the main consequences of the pathology. In addition, it was observed that intravitreal applications of proinflammatory cytokines increased the dilatation of blood vessels in the eye and this effect was reduced in the presence of AgNPs. Interestingly, application of AgNPs did not decrease the number of CD45/CD11b positive cells in the retina. This result suggests that AgNPs more likely regulate the activation process in microglia than the migration of immune cells into the retina.

The impact of AgNPs on immune cells has already been well documented. It has been shown that NPs influence the immune response in several ways depending on their size and coating [28–30]. These effects should be taken into consideration in the therapeutic application of AgNPs, where systemic suppression of the immune response could represent a potential risk. On the other hand, a local suppression of the inflammatory response during the pathological condition in the retina could prevent retinal cell damage and impairment of retinal function. There are several options for modification of AgNPs by coating them with a different capping agent. This method could improve some properties of NPs – like dissolution or stability, but also change the charge of the AgNPs [31–34]. It was also reported that AgNPs coated with poly vinyl pyrrolidone could cause angiogenesis [32]. This effect could be potentially harmful in the treatment of the ocular diseases. Thus, using this type of modifications should be well considered and tested. Altogether, our data suggest that the use of AgNPs for the treatment of retinal diseases could represent a promising therapeutic option.

## Data Availability

The experimental data that support the findings of this study are available in 10.5281/zenodo.13347988.

## Declarations

This study did not involve human participants. The experiments with animals were approved by the local Animal Ethics Committee of the Institute of Experimental Medicine of the Czech Academy of Sciences, Prague.
